# Optimization of the electrodeposition process of a polypyrrole/multi-walled carbon nanotube fiber electrode for a flexible supercapacitor

**DOI:** 10.1039/d2ra02430f

**Published:** 2022-06-21

**Authors:** Zhu Ping, Lian Junjie, Liu Yunchun

**Affiliations:** School of Instruments and Electronics, North University of China Taiyuan 030051 China

## Abstract

In the large-scale production of flexible supercapacitors, given the poor interface stability and the low mass loading of functional films on the fiber electrode, cyclic voltammetry (CV) and constant current (CC) electrodeposition methods were adopted to prepare polypyrrole/multi-walled carbon nanotubes (PPy/MWCNTs) on the surface of polyacrylonitrile (PAN) carbon cloth to explore the optimization process. The surface morphology and structural properties of the flexible electrode were characterized, and the electrical and electrochemical properties were studied. The research indicated that the PPy/MWCNTs were uniformly distributed on the fiber surface in the form of a linear structure and were amorphous and rich in carbon, nitrogen, and oxygen functional groups. A higher deposition current density helped improve the degree of coating of the MWCNTs with PPy and the number of oxygen-containing functional groups. The electrical and electrochemical properties of the flexible electrode prepared using the CC method were excellent; the electrochemical properties of the samples in the bent state were not significantly different from those in the straightened state. Using CC and CV methods, the conductivities of the samples were 32.4 S cm^−1^ and 24.1 S cm^−1^, the area-specific capacitance values were *C* 96.24 mF cm^−2^ and 46.18 mF cm^−2^ at a scan rate of 100 mV s^−1^, the equivalent series resistance *R*_s_ values were 2.74 Ω and 4.67 Ω, the specific capacitance retention rates were 94.4% and 88.3% after 1000 cycles, and the capacitance retention rates were 89.7% and 80.6% after 5000 cycles, respectively. The differences in the performances of the flexible electrodes using the same preparation solvent and different preparation processes were due to the higher deposition current density of the CC method compared with that of CV. The former enhanced the polymerization degree of the PPy/MWCNT flexible electrode and improved the electrochemical performance. The presented research results are significant for the optimization of large-scale production processes.

## Abstract 2

The difference between the electrical and electrochemical properties of PPy/MWCNTs flexible electrodes was found to be due to the constant current method having a higher deposition current density, leading to a higher degree of polymerization.

## Graphical abstract

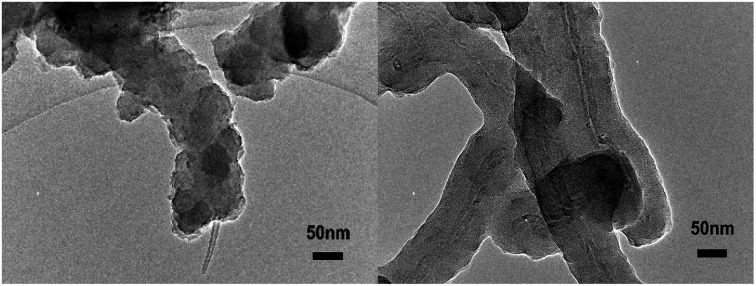

## 1 Introduction

Flexible fiber supercapacitors have received extensive attention in the field of flexible electronic fabrics.^1–6^ The elemental compositions of flexible fiber and ordinary supercapacitors are the same: a collector, electrode, electrolyte, and encapsulation material; simultaneously, owing to their fiber characteristics, they are different in terms of materials and assembly methods. The function of the fiber surface of a supercapacitor, the compositions of which are twisted pair, coaxial, and double electrode parallel type, plays the role of a flexible substrate and fluid collector.^7–9^ Mainly made of carbon and conductive polymers, electrode materials, such as carbon nanotube (CNT)/polyacrylonitrile (PAN) fibers, porous graphene (G)/CNT carbon fibers, porous Ni(OH)_2_/Ni films, CNT composite fibers, and amorphous MnO_2_ nanowires, are prepared by coating, electrophoretic deposition, etching processing combined with oxidation deposition, and electrodeposition on carbon cloth fibers or other flexible substrates.^10–14^ However, for carbon materials with low conductivity (conductivity is usually 10 to 10^4^ S cm^−1^) and conductive polymers (conductivity is usually 10^−3^ to 10^3^ S cm^−1^),^15^ there are two difficulties in preparing flexible electrode materials by coating, electrophoretic, and etching combined with oxidation deposition treatments. First, it is not easy to deposit oriented, continuous, stable electrodes directly on large-area fiber surfaces without using adhesives, which increases the internal resistance of flexible fiber supercapacitors, resulting in their low capacitance retention, poor cycling performance, low surge resistance, and other electrical performance problems.^16–20^ Second, electrode material stuck to the fiber surface of carbon cloth easily falls off due to the small diameter, water repellency, and low mechanical strength of the material, which causes a reduction in the mass load of the electrode material.^21–24^

Controlled by potential rather than by heat, the electrodeposition method regulates the doped state of the polymer efficiently and effectively in many preparation processes. It has been proven to be compatible with the most advanced semiconductor manufacturing technology and can be applied to energy storage micro-devices such as implantable devices or other microsystems.^25^ Therefore, it shows promising prospects for use in preparing high-quality electrode materials based on flexible substrates. However, Zhou *et al.*^26^ reported that the area-specific capacitance *C* of a Ni/rGO/MnO_2_ flexible electrode prepared by cyclic voltammetry (CV) was 37 mF cm^−2^ at a scan rate of 100 mV s^−1^. Hou *et al.*^27^ suggested that the area-specific capacitance *C* of polyaniline co-doped with a sulfuric acid and perchloric acid flexible electrode prepared using the constant current (CC) method was 140.2 mF cm^−2^ at a scan rate of 10 mV s^−1^. These studies show that flexible electrodes based on electrodeposition processes exhibit poor interface stability and low mass load.

One of the crucial ways to improve electrochemical performance is using porous structured materials as reinforcement carriers to prepare composite electrodes. Due to their excellent mechanical properties, aspect ratio, good thermal stability, and electrical conductivity, CNTs are often added as reinforcement materials to conductive polymers (*e.g.*, polypyrrole, polyaniline, polythiophene, and their derivatives) to improve electrochemical performance. However, the CNTs on the market are used directly after removing the catalyst, making the bonds between the CNTs and conductive polymers weak. The electrical performance of conductive polymer supercapacitors deteriorates after long charge/discharge cycles or storage, with the degradation of the materials,^28^ which is more extreme in electrodes with fiber substrates. Given the above problems, Zhao *et al.*^29^ grafted aniline (ANI) monomer on the surface of CNTs *via* organic synthesis, and then prepared CNTs/PANI composites using a CV method in a sulfuric acid solution containing ANI. The specific capacity was around 366 F g^−1^ at a single electrode mass of 27.27 mg and charge/discharge current of 8 mA, and the capacity decay was less than 5% after 200 cycles. While the above research method improves the compounding degree of two active substances with different properties, the cycling stability of the electrode is imperfect. The main reason for this imperfection is that the potential rate is in the form of a triangular wave in CV measurements after repeated scans, making the polymerization process of polymeric monomers on CNTs or other reinforcing materials uneven, resulting in a small and incompact nucleation structure of conductive polymer and the weak bonding of the interface between the electrode and the substrate. CC electrodeposition is a viable solution to ensure that monomers polymerize uniformly on CNTs or other reinforcing materials.

This study explores the effects of different preparation processes on the interfacial stability and mass loading of polypyrrole/multi-wall carbon nanotubes (PPy/MWCNTs) electrodeposited on a polyacrylonitrile (PAN) carbon cloth surface using CV and CC methods, respectively. The effects of CV and CC methods on the electrical and the electrochemical properties of PPy/MWCNTs flexible electrodes were analyzed in terms of conductivity, apparent morphology, physical properties, area-specific capacitance, AC impedance, galvanostatic charge/discharge performance, and cyclic stability to provide basic parameters for the process optimization of their large-scale production.

## 2 Experimental

### 2.1 Materials

The experimental materials included: MWCNTs (diameter of 30–50 nm, length of 10–30 μm, Beijing Boyu Hi-Tech New Material Technology Co., Ltd.), pyrrole (Py, analytical purity), sodium dodecyl benzene sulfonate (SDBS, analytical purity), sodium chloride (NaCl, analytical purity), and PAN carbon cloth (W0S1009, thickness 0.33 mm, Taiwan Carbon Energy Co.).

Because the surface of the MWCNTs is inert, easily agglomerated, and difficult to disperse, it is often necessary to chemically modify the surface of MWCNTs before preparing composites. The anionic surfactant SDBS was used to effectively prevent MWNT aggregation. Therefore, the electrolytic solution was prepared as follows: 10 mL of deionized water, 100 μL of pyrrole, 100 μL of SDBS solution (0.023 mol L^−1^), 10 mg of carboxylated MWCNTs, sonicated for 10 min, at pH 3.5–4.5.

### 2.2 Flexible electrode preparation

The flexible electrode material was PAN carbon cloth, soaked in concentrated nitric acid for 30 min, ultrasonically cleaned with deionized water, and dried naturally for 24 h, which then was made into several 1 cm × 3 cm pieces as samples.

A three-electrode system was formed using carbon cloth as the anode, platinum wire as the counter cathode, and a saturated calomel electrode as the reference electrode. The flexible electrodes were prepared using a CHI660C electrochemical workstation *via* CV and CC methods, respectively.

The CV method parameters were set as follows. The scan rate was 100 mV s^−1^ over a voltage window of −0.4 to 1.0 V at 25 °C for 56, 100, 150, and 200 scans, respectively. The flexible electrode samples 1#-1, 1#-2, 1#-3, and 1#-4 were prepared, dried, and treated in an air atmosphere at 65 °C for 1 h, and then stored for use.

The CC method parameters were set as follows. The electrodeposition currents/polymerization times were 6 mA/1200 s, 6 mA/1800 s, 10 mA/1800 s, and 20 mA/1800 s at 25 °C. The flexible electrode samples 2#-1, 2#-2, 2#-3, and 2#-4 were prepared, dried in an air atmosphere at 65 °C for 1 h, and then stored for use.

### 2.3 Tests

The flexible electrodes were wetted for more than 24 h at 20 °C and 65% humidity. A Zc-90g high insulation resistance measuring instrument was used to measure the resistance of 2 cm of yarn in the flexible electrode. Each sample was tested at five positions, and the conductivity of the flexible electrode was calculated using eqn (1):

1 *σ* = *L*/(*R*·*S*)

where *σ* (S cm^−1^) is the conductivity, *L* (cm) is the yarn length, *R* (Ω) is the yarn resistance, and *S* (cm^2^) is the cross-sectional area of the yarn.

The surface morphology of the flexible electrodes was observed by scanning electron microscopy (SEM, JSM-6700F) and transmission electron microscopy (TEM, JEM-2010), and the crystalline phase and structure of the samples were analyzed by X-ray diffractometry (XRD, MAX-2400). X-ray photoelectron spectroscopy (XPS) was used to probe the surface composition of the samples, and the chemical states were studied by X-ray photoelectron spectroscopy (XPS). The CV, AC impedance, and CC charge/discharge characteristics of the samples were tested in 1 mol L^−1^ NaCl electrolyte using a CHI660C electrochemical workstation with a three-electrode system. The area-specific capacity (*C*, mF cm^−2^) was derived from the CV curves to determine the performance of the flexible electrodes according to eqn (2):^30^2
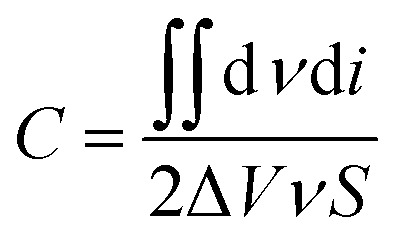


In this equation, 
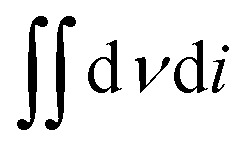
 is the area of the CV curve, Δ*V* (*V*) is the scanning voltage range, *ν* (V s^−1^) is the scan rate, and *S* (cm^2^) is the effective area of the flexible electrode.

## 3 Results and discussion

### 3.1 Conductivity

Many factors such as dopant, medium selection, reaction temperature (*T*), pH value, and voltage/current affected the polymerization degree of PPy in the electrodeposition process. The voltage/current of these factors was the easiest to use to realize automatic control in the process of large-scale production, for the differences between voltage/current waveform shapes or numerical values could make a difference in terms of conductivity by several orders of magnitude. Hence, the conductivity of the flexible electrode was measured to investigate its conductivity.

The conductivities of the 1# sample series were 17.6, 24.1, 21.4, and 18.3 S cm^−1^, respectively. The conductivities of the 2# sample series were 22.3, 27.5, 32.4, and 21.6 S cm^−1^, respectively. The highest conductivity of the 1#-2 flexible electrode prepared using the CV method was 24.1 S cm^−1^ after 100 cycles. The conductivity of the flexible electrode decreased after 150 and 200 cycles. Therefore, the 1#-2 sample was simplified as 1# for further research. The highest conductivity of the 2#-3 flexible electrode using the CC method was 32.4 S cm^−1^ at an electrodeposition current/polymerization time of 10 mA/1800 s. When the electrodeposition current was 20 mA, the conductivity of the flexible electrode decreased. The 2#-3 sample was therefore simplified as 2# for further research.

Before the electrochemical testing, the conductivity of the 2# flexible electrode was significantly higher than that of the 1# sample, which was attributed to the formation of more π–π noncovalent bonds in the hybrid system under the application of CC, resulting from the uniform dispersion of carboxylated MWCNTs in the PPy matrix. After the electrochemical testing, the conductivity of the two flexible electrodes decreased, in which the conductivity of the 1# flexible electrode decreased to 18.5 S cm^−1^ and that of the 2# flexible electrode to 29.7 S cm^−1^. The conductivity of the 2# flexible electrode decreased to significantly lower than that of 1#, which was attributed to the dense nucleation structure of PPy and the better adhesion between the PPy/MWCNTs and carbon cloth fiber.

### 3.2 Morphology characterization

**Fig. 1:**
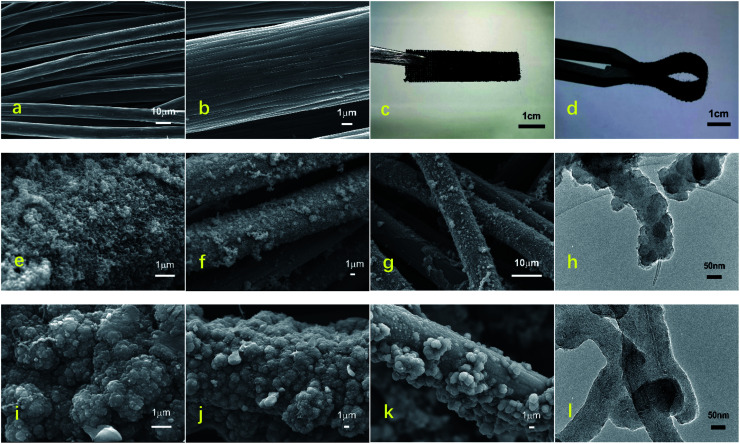
Schematic illustration of the morphologies of the flexible electrodes. (a) and (b) Optical images of the flexible fibers without deposited functional materials, (c) and (d) optical images of the 1# and 2# samples after preparing functional materials, (e) and (f) SEM images of the 1# sample, (g) SEM image of the 1# sample after electrochemical testing, (h) TEM of the 1# sample, (i) and (j) SEM images of the 2# sample, (k) SEM image of the 2# sample after electrochemical testing, and (l) TEM image of the 2# sample.

Fig. 1(a)–(d) show the overall morphology of the carbon cloth before and after the electrodeposition of the PPy/MWCNTs. The SEM images under low magnification showed that the carbon cloth consisted of smooth fibers with diameters of around 15–17 μm (Fig. 1(a)), and grooves with sizes of 200–400 nm were observed on the surface of a single carbon cloth fiber under high magnification (Fig. 1(b)). The surface of carbon cloth fibers without electrode material deposition was smooth and flexible. After the carbon cloth had been covered with the PPy/MWCNT electrode material, the carbon cloth became thicker and rougher. However, its flexibility was still excellent (Fig. 1(c) and (d)), which indicated that the interface between the flexible fiber surface and the electrode material was well bonded.

The macrostructure and microstructure of the carbon cloth fiber influenced the conductivity of the flexible electrodes. From a macroscopic aspect, the flexible electrode had a uniform structure and good continuity and was closely bonded with the interface of the carbon cloth fiber, which provided excellent movement channels for the carriers and endowed the flexible electrode with superb conductivity. From a microscopic aspect, the structural morphology of the 1# sample prepared using the CV method was a fluffy and porous flocculent, as shown in Fig. 1(e)–(g). The flexible electrode structure was relatively uniform. The adhesion with carbon cloth fiber was not tight, and severe detachment occurred after electrochemical testing (Fig. 1(g)). Therefore, the interface stability of the 1# sample was poor.

Furthermore, TEM was used to observe more structural information of the 1# sample (Fig. 1(h)). It showed that MWCNTs were partially covered with PPy, the surface of the wire-like structure was uneven, and the overall appearance was spring-like. From Fig. 1(i)–(k), the structural morphology of the 2# sample showed a protruding granular or cauliflower-like structure. It was uniform and dense, the flexible electrode appeared partially detached after the electrochemical tests, and the interfacial stability was excellent overall. This indicated that the PPy was completely covered with MWCNTs, from the TEM of the 2# sample (Fig. 1(l)). The wire-like structure had a uniform diameter and smooth surface, and the overall appearance was intertwined.

The electrical conductivity of the flexible electrodes is closely related to the degree of polymerization of functional materials. During the electrodeposition process, Py polymerization centered on the MWCNTs, around which pyrrole radical ions grew in a tight arrangement. In addition, the MWCNTs also acted as interlayer pillars of PPy, preventing the spontaneous collapse of the latter on the surface fibers. Since the CC method has a more significant deposition current density and CC value than the CV method, the pyrrole entered the three-dimensional network of the MWCNT layers and the interior of the carbon cloth and polymerized uniformly. It increased the degree of polymerization and the density of the PPy nanoparticles in the electrode and the surface coverage of the carbon cloth fibers, thus increasing the mass loading rate, resulting in better interfacial stability of the electrode structure. The above analysis also supported the results of the conductivity tests.

### 3.3 Structural characterization

**Fig. 2:**
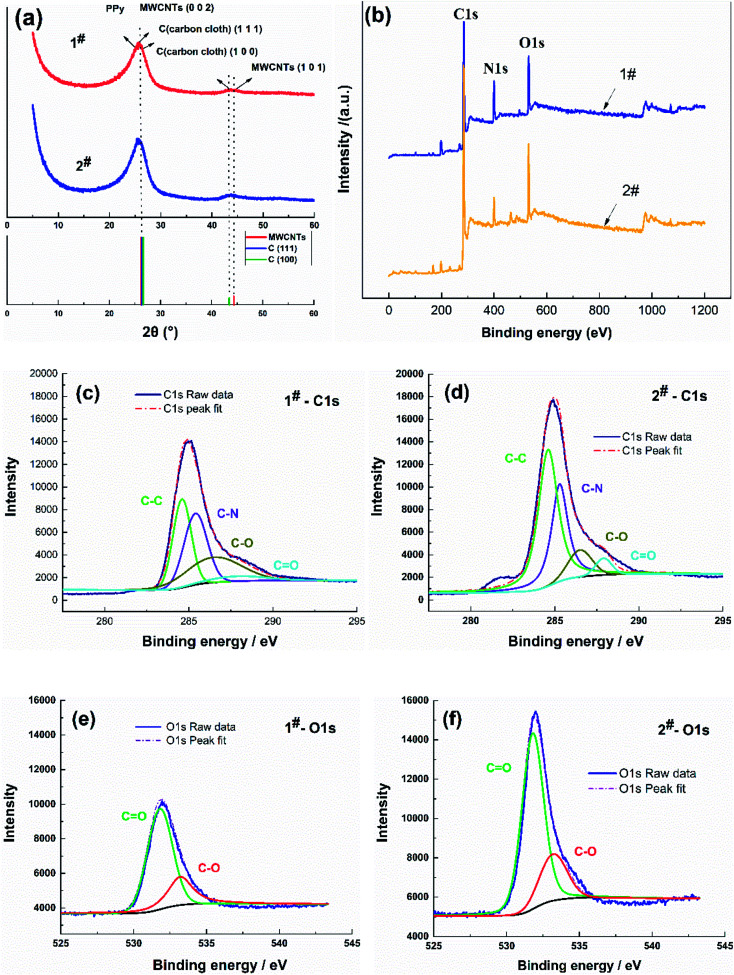
XRD patterns and XPS spectra of the flexible electrodes. (a) XRD patterns, (b) survey XPS spectra, and (c) 1# C1s, (d) 2# C1s, (e) 1# O1s, and (f) 2# O1s XPS spectra.

The PPy/MWCNTs flexible electrodes were further researched using XRD and XPS, as shown in Fig. 2.

XRD patterns corresponding to the 1# and 2# samples show diffraction peaks at 2*θ* = 26° and 44.6° (database: ICSD Patterns), as shown in Fig. 2(a). The diffraction peak profile at 2*θ* = 44–45° corresponded to the C (101) of the MWCNTs and C (100) of the carbon cloth, confirming the characterization of the MWCNTs. The diffraction peak profile at 2*θ* = 26° was broad and intense, which was attributed to the stacking of the diffraction peaks C (111) of the carbon cloth (2*θ* = 25.4°), the diffraction peak of amorphous polypyrrole (2*θ* = 21.1°), and the corresponding diffraction peak C (002) of the MWCNTs (2*θ* = 26.8°).^31^ This showed that the prepared PPy/MWCNTs were amorphous and had no crystalline form. The diffraction peaks at 2*θ* = 26° for the PPy/MWCNTs flexible electrode prepared using the CC method were more intense than those of the electrode prepared using the CV method, indicating that the flexible electrode prepared using the CC method exhibited better polymerization.

In Fig. 2(b), the XPS spectra of the 1# and 2# samples show three typical peaks corresponding to the binding energies of C1s, N1s, and O1s.^32,33^ XPS analysis indicated no significant difference in the intensities of the N1s and O1s peaks among the samples, and the C1s peak intensities of the 2# sample were significantly higher than those of 1#. The high-resolution C1s spectra were deconvoluted into four individual peaks (Fig. 2(c) and (d)) located at 284.6 eV, 285.4 eV, 286.5 eV, and 287.9 eV, corresponding to C–C, C–N, C–O, and C=O, respectively. The 1# and 2# samples showed that the dominant C–C and C–N peak intensity suppressed the C–O and C=O peaks, which indicated that the structure was dominated by carbon sp^2^ hybridization, consistent with the XRD results. The C–C and C–N peak intensity of the 2# sample was more intense than that of the 1# sample, suggesting an increase in PPy aggregation. The high-resolution O1s spectra were further deconvoluted into two peaks (Fig. 2(e) and (f)), which were attributed to oxygen in the carbonyl group C=O (at 531.8 eV) and oxygen in the phenol and lactone groups C–O (at 533.2 eV). The C=O and C–O peak intensity of the 2 # sample was stronger than that of the 1# sample, which also indicated that the CC method had a higher current density, improving the PPy/MWCNT polymerization effect and increasing the content of oxygen-containing functional groups on the surface of the material, which helped to improve the performance of the flexible supercapacitors. These results showed that PPy/MWCNTs were successfully prepared on carbon cloth fibers.

### 3.4 Electrochemical characterization

**Fig. 3:**
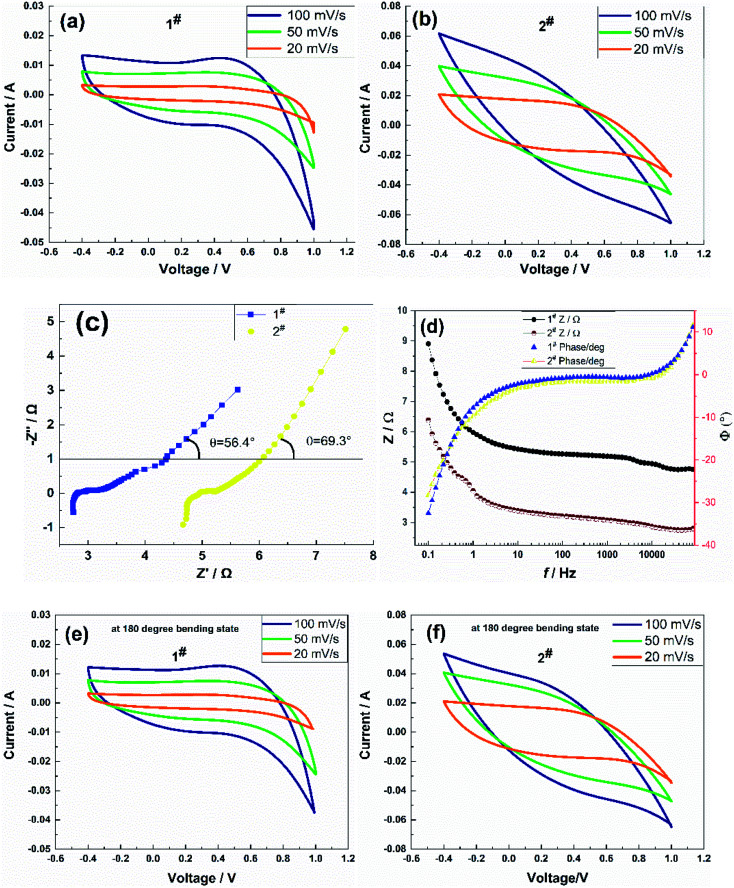
(a) and (b) Cyclic voltammograms, (c) AC impedance spectra, (d) impedance Bode diagrams of the flexible electrodes in the stretched state, and (e) and (f) cyclic voltammograms of the flexible electrodes in the bent state.

The electrochemical performance testing adopted a three-electrode system, and the potential scan range was set at −0.4 to +1 V. When the scan rate was 20 mV s^−1^, 50 mV s^−1^, and 100 mV s^−1^, the CV curve and impedance spectrum of the sample in its straight form and the CV curve of the sample in its bent form were recorded, as shown in Fig. 3.

The symmetry of CV curves in Fig. 3(a) and (b) showed that the 1# sample deviates obviously from symmetrical, which was possibly due to the large internal resistance of the sample causing the slow transport rate of electrolyte ions in the matrix, resulting in a response lag in the charging and discharging process. The CV curves of the 2# sample showed better reversibility redox in an aqueous NaCl solution. It could be calculated from eqn (2) that the area-specific capacitance was related not only to the current magnitude but also to the area enclosed by the CV curves at a constant scan rate. Obviously, under the three scan rates, the area surrounded by the corresponding curve of the two samples at 100 mV s^−1^ was the largest, at 50 mV s^−1^ the second, and at 20 mV s^−1^ the smallest, which also indicated the good multiplicity of the flexible electrode. The area-specific capacitances were 62.17 mF cm^−2^, 57.71 mF cm^−2^, and 46.18 mF cm^−2^ for 1# sample, and 273.41 mF cm^−2^, 162.06 mF cm^−2^, and 96.24 mF cm^−2^ for 2# sample at an effective area of 2 cm^2^ of the flexible electrode, respectively. The area-specific capacitance values at a scan rate of 100 mV s^−1^ of both samples were higher than that of the Ni@rGO@MnO_2_ flexible electrode (*C* = 37 mF cm^−2^) reported in the literature,^26^ among which the capacitance performance of the 2# sample prepared using the CC method was better than that of the CV method. The frequency response of the flexible electrodes was measured *via* the AC impedance method.

A small-amplitude sinusoidal AC signal of 5 mV was applied to the samples, and the frequency range was set to 0.1–100 kHz. Fig. 3(c) shows the impedance spectra of the samples. The impedance spectra showed semicircular arcs in the high-frequency region and straight lines in the low-frequency region, which confirmed that the samples were hybrid supercapacitors with a combination of pseudocapacitor and double-layer capacitor characteristics. The equivalent series resistance *R*_s_ of the 1# sample was 4.67 Ω, the contact resistance *R*_ct_ was 0.57 Ω, and the angle *θ* between the straight line and the real axis in the low-frequency part *θ* was 69.3°. The equivalent series resistance *R*_s_ of the 2# sample was 2.74 Ω; the contact resistance *R*_ct_ was 0.6 Ω; and the angle *θ* between the straight line of the low-frequency part and the real axis was *θ* = 56.3°. The contact resistance *R*_ct_ of the two samples was significantly lower than that of the PANI/SWCNT flexible electrode (*R*_ct_ = 19.9 Ω) reported in the literature,^23^ which indicated that the samples exhibited better conductivity, effectively reducing the voltage drop of the supercapacitor during discharge and improving the anti-surge capability. The 2# sample exhibited a lower equivalent series resistance *R*_s_ than the 1# sample in the high-frequency region, and the contact resistance *R*_ct_ was slightly higher than for the 1# sample, which suggested that the pseudocapacitance of the 2# sample was more significant than that of the 1# sample and had better charge transfer performance and a faster ion diffusion process. These phenomena were possibly caused by the significant difference in microscopic morphology or pore structure of the flexible electrode structure of the samples. The above analysis also corroborated the conductivity, as well as the CV test results.

Fig. 3(d) shows the impedance baud diagrams of the two samples, both of which were close to that of an ideal hybrid supercapacitor; there were no significant differences in the phase angles of both samples. When the sample frequency was lower than 10 Hz, the phase angle gradually increased; when it was higher than 10 Hz, the phase angle gradually decreased, and the change in the phase angle was the same in the range of 20–10 kHz *Φ* = 0°, indicating that the flexible electrode could be approximated as a resistive element at middle and high frequencies. The impedance amplitude of the samples showed little change in the range of 20–10 kHz; it increased rapidly with a decrease in frequency below 10 Hz, and the impedance amplitude of the 2# sample was slightly lower than that of the 1# sample. The operating frequency range of both samples was wide, and the frequency characteristics of the 2# sample were slightly better than those of the 1# sample.

Fig. 3(e) and (f) show the CV curves of the sample in the bent 180° state after 50 cycles. The CV curves of samples in the bent state were slightly different from those in the straightened state, but the area in the curves did not change much. At scan rates of 20 mV s^−1^, 50 mV s^−1^, and 100 mV s^−1^, the area-specific capacitances of the 1# sample were 60.85 mF cm^−2^, 54.75 mF cm^−2^, and 44.78 mF cm^−2^, respectively, and those of the 2# sample were 276.15 mF cm^−2^, 165.31 mF cm^−2^, and 101.57 mF cm^−2^, respectively. The specific capacitance of the samples in the bent state was not significantly different from that in the stretched state at the three scan rates, in which the area-specific capacitance of the 2# sample was slightly higher, which suggested that the interface between the flexible electrode and the carbon cloth fiber prepared using the CC method exhibited stronger adhesion and better flexibility.

**Fig. 4:**
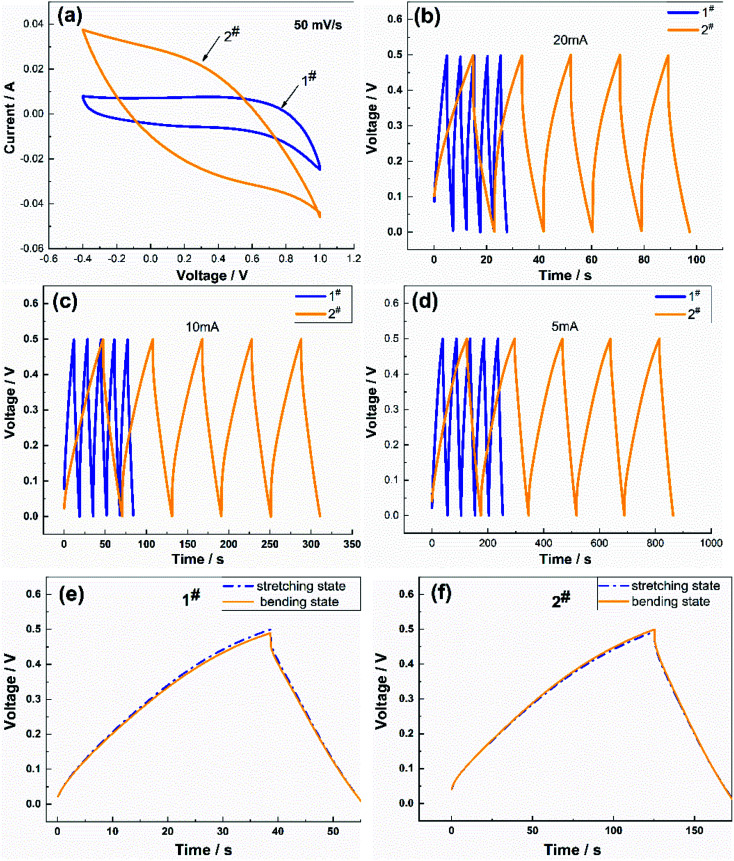
(a) Comparison of the cyclic voltammetry tests of the flexible electrodes at a scan rate 50 mV s^−1^; (b), (c), and (d) comparison of the charge/discharge data under different charging currents; and (e) and (f) comparison of the charge/discharge data at 5 mA between the stretched and bent states.

Fig. 4 shows a comparison of the charge/discharge performance of the two samples. Fig. 4(a) presents the CV curves of the samples at a scan rate of 50 mV s^−1^. The CV curves of the two samples were very different, where the CV curve of the 2# sample had a shuttle shape with a larger area, which meant that the charging and discharging time was longer. The charging and discharging characteristics of the two samples (Fig. 4(b)–(d)) were compared at charging currents of 5 mA, 10 mA, and 20 mA, respectively, and the discharge curves of the samples were symmetrical in shape. The decrease in the coulombic efficiency was not noticeable, which indicated that the reversibility of the charging and discharging of the flexible electrode was excellent and the capacitance was suitable, where the specific capacitance of the 2# sample was more significant than that of the 1# sample, and the charging and discharging time longer than that of the 1# sample. These differences in performance were attributed to the lower specific capacitance of the flexible electrode prepared using the CV method, which was due to the lower number of oxygen-containing functional groups in the soft and porous flocculent electrode material. Therefore, the electrode material attached to the surface of the flexible fibers needed to be uniform and compact to contribute to the capacitive performance. The charging and discharging characteristics of the two samples between the stretching state and bent state were compared at a charging current of 5 mA (Fig. 4(e) and (f)), and the discharge curves were symmetrical in shape. The charging and discharging characteristics of the samples in the bent state were not significantly different from those in the stretched state.

**Fig. 5:**
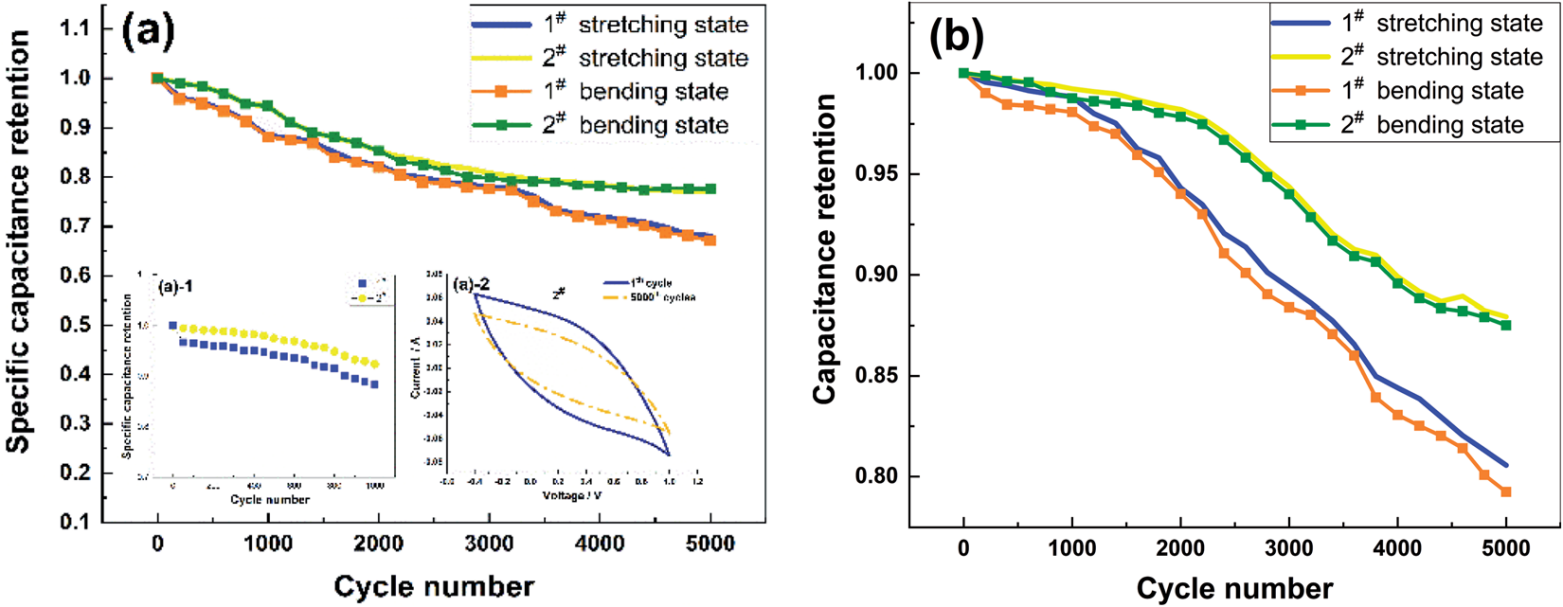
Comparison of the cycling stability of the flexible electrodes. (a) Comparison of the specific capacitance retention of the 1# and 2# samples after 5000 cycles and (b) comparison of the capacitance retention of the 1# and 2# samples after 5000 cycles.

Fig. 5 shows a comparison of the cycling stability of the two samples, which were tested using CV at a scan rate of 100 mV s^−1^. The specific capacitance retentions of the 1# and 2# samples were 96.07% and 99.11% after 200 cycles and 88.3% and 94.4% after 1000 cycles, respectively (Fig. 5(a)-1); those of the 2# sample were significantly better than the values of a CNT/PANI composite reported in the literature^29^ (95% specific capacitance retention after 200 cycles). The specific capacitance retentions of the 1# and 2# samples decreased to 68.1% and 77.2% after 5000 cycles (Fig. 5(a)). The specific capacitance retentions of the samples in the bent state were not significantly different from those in the stretched state. However, changes in the CV curve shapes were not apparent (Fig. 5(a)-2), possibly due to the insignificance of the degradation of the PPy and related to the decrease in the thickness of the PPy/MWCNT films after a long cycling period. The capacitance retentions of the 1# and 2# samples were 80.6% and 89.7% after 5000 cycles, respectively (Fig. 5(b)). There was no significant difference between the capacitance retention rate of sample 2# in the bent state and that in the stretched state. However, the capacitance retention of sample 1# in the bent state showed a downward trend. After 5000 cycles, the capacitance retention of sample 1# in the bent state decreased to 98.4% in the stretched state. The capacitance retentions of the 1# and 2# samples were significantly better than the specific capacitance retentions, and the capacitance retention of the 2# sample was considerably better than that of 1#. Cycling stability experiments showed that the CC method was more suitable for PPy/MWCNT flexible electrode preparation.

## 4 Conclusion

The flexible electrodes were produced by electrodepositing the PPy/MWCNT material on the surface of PAN carbon cloth fibers *via* CV and CC methods, respectively, and the research showed that:

(1) The conductivity of the flexible electrode prepared using the CC method was better than that of CV before and after the electrochemical tests. Before the electrochemical tests, the highest conductivities *σ* of the flexible electrodes prepared using the CV and the CC methods were 24.1 S cm^−1^ and 32.4 S cm^−1^, respectively. After electrochemical testing, the conductivity decreased to 18.5 S cm^−1^ and 29.7 S cm^−1^, respectively.

(2) SEM, TEM, XRD, and XPS analysis indicated that the flexible electrode prepared using the CV method exhibited a fluffy and porous flocculent shape, PPy partially covered the MWCNTs, and the interfacial stability with the carbon cloth fibers did not function effectively. The flexible electrode prepared using the CC method exhibited a raised cauliflower shape, PPy completely covered the MWCNTs, and the interfacial stability with the carbon cloth fibers was better overall. Therefore, the PPy/MWCNT flexible electrode polymerization prepared using the CC method was better. The PPy/MWCNTs were amorphous and rich in carbon, nitrogen, and oxygen functional groups. The higher deposition current density helped to improve the degree of coating of the MWCNTs with PPy and the number of oxygen-containing functional groups.

(3) In the electrochemical performance tests, the flexible electrode prepared using the CC method exhibited the best electrochemical performance. The area-specific capacitance of the flexible electrode prepared using the CV method was 46.18 mF cm^−2^ at a scan rate of 100 mV s^−1^. The flexible electrode prepared using the CC method was 96.24 mF cm^−2^ in its stretched state. There was no significant difference between the area-specific capacitance/capacitance retention of the two samples in the bent state and in the stretched state; the equivalent capacitances of the flexible electrodes prepared using the CV and CC methods were 4.67 Ω and 2.74 Ω, respectively. The series resistance *R*_s_ and contact resistance *R*_ct_ were 4.67 Ω and 2.74 Ω, and the angle *θ* between the straight line and the real axis of the low-frequency part were 69.3° and 56.3°. Hence, the flexible electrode prepared using the CC method exhibited better anti-surge capability and frequency characteristics. After 1000 cycles of voltammetry tests, the specific capacitance retention rates of the flexible electrode prepared using the CV and CC methods were 88.3% and 94.4%, decreasing to 68.1% and 77.2% after 5000 cycles, with capacitance retention rates of 80.6% and 89.7% after 5000 cycles.

(4) Using the same preparation solvent and different preparation processes, the significant difference observed between the electrical and electrochemical properties of the PPy/MWCNT flexible electrodes was because the CC method had a higher deposition current density, leading to a higher degree of polymerization than when the CV method was used. This ensured the uniformity and tightness of the electrode material attached to the surface of the flexible fibers, thus guaranteeing interfacial stability and mass loading, improving the electrochemical performance of the flexible supercapacitors.

## Conflicts of interest

The authors declare that they have no known competing financial interests or personal relationships that could have appeared to influence the work reported in this paper.

## Supplementary Material
